# Retention and use of newborn resuscitation skills following a series of helping babies breathe trainings for midwives in rural Ghana

**DOI:** 10.1080/16549716.2017.1387985

**Published:** 2017-10-23

**Authors:** Darren Eblovi, Patricia Kelly, Georgina Afua, Sarah Agyapong, Siddhartha Dante, Matthew Pellerite

**Affiliations:** ^a^ Department of Pediatrics, Northwestern University Feinberg School of Medicine, Chicago, IL, USA; ^b^ Department of Global Health, University of Colorado Hospital, Aurora, CO, USA; ^c^ Department of Midwifery, Ghana Health Service, Sunyani, Ghana; ^d^ Department of Medicine, University of Chicago Medical Center, Chicago, IL, USA; ^e^ Department of Pediatrics, University of Chicago Medical Center, Chicago, IL, USA

**Keywords:** Helping babies breathe, neonatal resuscitation, maternal child health, intrapartum-related neonatal death, health education

## Abstract

**Background**: The Helping Babies Breathe (HBB) program teaches basic newborn resuscitation techniques to birth attendants in low-resource settings. Previous studies have demonstrated a decrease in mortality following training, mostly in large hospitals. However, low-volume clinics in rural regions with no physician immediately available likely experience a greater relative burden of newborn mortality. This study aimed to determine the impact of HBB trainings provided to rural Ghanaian midwives on their skills retention and on first 24 hour mortality of the newborns they serve.

**Methods**: American Acadamy of Paediatrics (AAP)-trained Master Trainers conducted two 2-day HBB trainings and 2-day refresher courses one year later for 48 midwives from Ghanaian rural health clinics. Trainee skills were evaluated by Objective Structured Clinical Examination (OSCE) at three time points: immediately after training, four months after training, and four months after the refresher. Midwives recorded the single highest level of resuscitation performed on each newborn delivered for one year.

**Results**: 48 midwives attended the two trainings, 32 recorded data from 2,383 deliveries, and 13 completed OSCE simulations at all three time points. The midwives’ OSCE scores decreased from immediately after training (94.9%) to four months later (81.2%, p < 0.00001). However, four months following the refresher course, scores improved to the same high level attained initially (92.7%, p = 0.0013). 5.0% of neonates required bag-mask ventilation and 0.71% did not survive, compared with a nationwide first 24 hour mortality estimate of 1.7%.

**Conclusions**: The midwives’ performance on the simulation exercise indicates that an in-depth refresher course provided one year after the initial training likely slows the decay in skills that occurs after initial training. Our finding that 5.0% of newborns required bag-mask ventilation is consistent with global estimates. Our observed first 24 hour mortality rate of 0.71% is lower than nationwide estimates, indicating the training likely prevented deaths due to birth asphyxia.

## Background

In 1990, the United Nations targeted child mortality with Millennium Development Goal 4, which aimed to decrease mortality of children less than five years of age by two thirds. Since that time, despite an increased birth rate, the annual number of child deaths has decreased. The World Health Organization (WHO) estimates deaths in children under five decreased from 9.9 million in 2000 to 6.3 million in 2013 [1]. However, the mortality rate in children aged 1–59 months declined more significantly than did mortality for neonates, with a resultant increase in the contribution of neonatal deaths to the under-five mortality rate. As of 2013, intrapartum-related neonatal death (birth asphyxia) remained the cause of at least 662,000 deaths per year [1].

With the goal of preventing these deaths, the American Academy of Pediatrics (AAP) and its global partners created the Helping Babies Breathe (HBB) program, which teaches newborn resuscitation techniques to birth attendants in low-resource settings. The WHO estimates that while 5–10% of newborns worldwide require some level of resuscitation to initiate respirations, including 3–6% who require bag-mask ventilation, less than 0.1% of neonates require advanced resuscitation techniques such as chest compressions and vasoactive medications [2]. Furthermore, infants requiring such efforts to survive the immediate post-delivery period would likely require ongoing support not readily available in regions targeted by HBB. Subsequently, HBB was designed to teach proper drying, stimulation, clearing the airway as needed, and bag-mask ventilation, without teaching the advanced techniques of chest compressions and medication administration.

Since its inception in 2010, several studies have examined the impact of HBB training on trainee skills. The program includes a validated simulation evaluation tool called an Objective Structured Clinical Examination (OSCE) [3,4]. Several studies have demonstrated trainee improvement on the examination using a pre-post design [5–8]. However, evidence also suggests performance decreases significantly over time when learners are not provided with ongoing refresher training [9,10]. Research has demonstrated improvement in skills after various types of refresher courses [7–10], but consensus has yet to be reached for the ideal timing and duration for such education.

In addition to outcomes relating to skills retention, previous research has also demonstrated an association between HBB training and either decreased newborn mortality rate or decreased fresh stillbirth rate (suggesting many apneic newborns were improperly labeled as stillborn) [11–15]. However, although some of these studies included low-volume rural clinics, the majority of births evaluated in these studies occurred in hospitals or larger clinics staffed full-time by physicians. Due to a higher relative birth rate and less direct involvement of physicians in the labor and delivery process, rural areas served only by small clinics suffer a greater burden of neonatal mortality from birth asphyxia [1,2].

This study has two principal aims: the first is to evaluate the retention of skills acquired by midwives working in small rural health clinics in Ghana following an initial HBB training compared with after an in-depth refresher course provided one year later. The second is to attempt, in a study population with limited retrospective data available, to evaluate the impact of the trainings on the first 24 hour mortality of the newborns the midwives serve.

## Methods

AAP-trained HBB Master Trainers from the non-profit organization Project C.U.R.E. conducted two Helping Babies Breathe trainings for 24 students each in the Brong-Ahafo and Eastern regions of Ghana. The Ghanaian Ministry of Health invited all midwives in rural health clinics in the areas surrounding the regional capitals to be the students, and the first 24 midwives to volunteer were included in the training.

The Brong-Ahafo and Eastern regions of Ghana are low-income areas with primarily agricultural economies. The majority of healthcare providers practice in hospitals in the regional capitals of Sunyani and Koforidua. There are no full-time physicians staffing the rural health clinics located in the surrounding villages.

Directors of the rural health clinics are physician’s assistants who report to a regional medical director. In addition to the clinic director, each clinic included in our study employs one or two midwives who perform an average of 5–30 deliveries per month. Midwives are managed by a regional midwife instructor, who visits the clinics several times per year to provide supervision and ongoing education.

To become a midwife in Ghana, high school graduates must first earn a three-year nursing degree, followed by an additional two years of midwifery training. However, midwives who were trained more than 10–12 years ago may have earned their credentials with fewer total years of study. Despite their training in midwifery, none of the students trained by HBB for this study had previous formal training in newborn resuscitation or possessed bag-mask ventilation devices before enrolling in the HBB course.

The trainings were consistent with the standardized Helping Babies Breathe curriculum: students were provided with two 8 hour sessions including introduction of the material, discussion, hands-on instruction, and repetitive practice of resuscitation techniques using NeoNatalie® mannequins (Laerdal Medical) with various clinical scenarios. Trainings were held in English. Although Ghanaians from the Brong-Ahafo region primarily speak Twi and those in the Eastern region speak Akan, all formal education nationwide is conducted in English, and therefore all of the study’s midwives were proficient.

Upon completion of training, trainers evaluated the midwives’ skills with a written test and two Objective Structured Clinical Examinations (OSCEs). The first (A) simulates the delivery of a healthy infant and the second (B) presents a late preterm infant born in secondary apnea who requires positive pressure ventilation to survive. Scenario B is scored out of 18 points. A score of 14, in addition to the completion of all four ‘critical steps’ (recognizes baby is not breathing, ventilates at 40 breaths per minute, looks for chest movement, and performs the five steps to improve ventilation) is required to pass.

Four months after the initial trainings, pediatricians that were also HBB Master Trainers visited the trainees in their clinics of employment. The pediatricians re-evaluated the midwives on the OSCE scenario (B) that requires bag-mask ventilation, using the same grading scale as after training. Only scenario B was used because it evaluated all of the skills required to complete scenario A. Following the simulation, the pediatricians provided the midwives with feedback on their performance and reviewed key skills.

**Figure 1.**
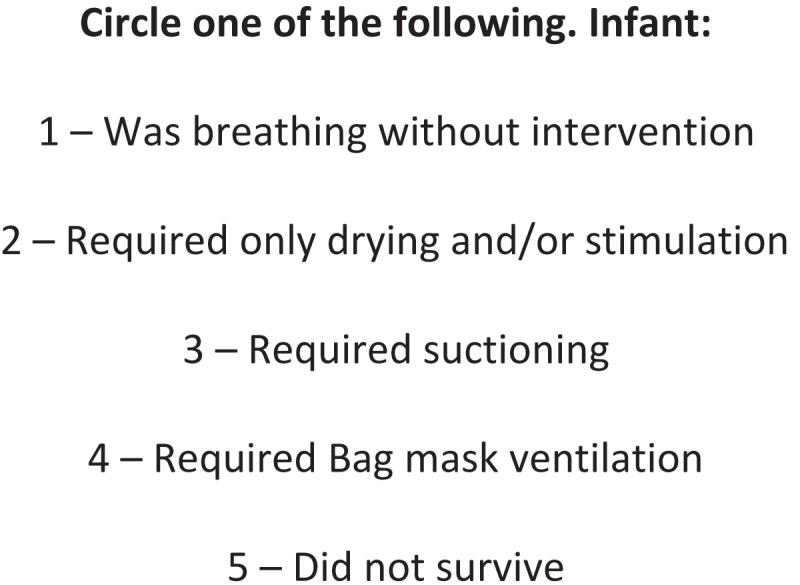
Delivery log.

The midwives were provided with delivery logs and instructed to record the single highest level of resuscitation they performed at each delivery attended using the format seen in Figure 1. As HBB teaches birth attendants to attempt resuscitation on all newborns, it was assumed that those who did not survive were provided with all possible interventions including bag-mask ventilation. Since receiving ventilation was the principal intervention in question, drying and stimulation were combined in order to make the logbooks more concise.

Participating health clinics generally discharge mothers and healthy newborns at 6 hours of life, but will observe less stable newborns for up to 24 hours. Newborns requiring ongoing support can be transferred to a higher level of care. Therefore, if the midwife recorded that the infant survived, it was assumed that the infant survived the first 24 hours of life, but not necessarily beyond.

The midwives collected data on all deliveries they attended for a one year period after the physicians’ visits. One year after the initial training, the midwives received a two day refresher course provided by the same instructor as the original training. The refresher training included the course ‘Essential Care for Every Baby,’ the course following HBB in the AAP’s Helping Babies Survive curriculum.

Sixteen months after the initial training (four months after the refresher course), the physician Master Trainers visited the midwives to repeat the OSCE. Delivery data recorded from the previous year were collected. Paired t-tests were used to compare mean scores achieved on the OSCE at the three time periods (immediately post-training, four months after training, and four months after refresher training).

We compared the rate of mortality in the first 24 hours of life documented by the midwives during the one year study period with nationwide estimates of first 24 hour mortality in Ghana by the World Health Organization from 2007–2013 [16].

## Results

**Figure 2.**
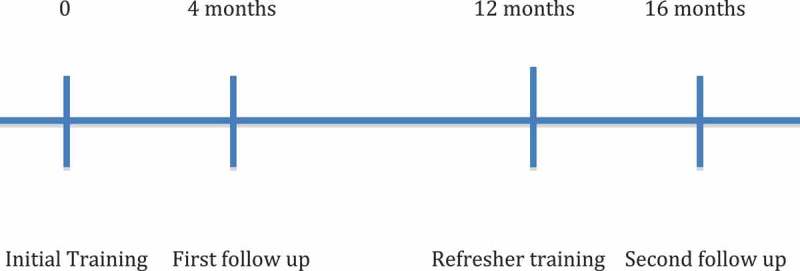
Study timeline.

**Figure 3.**
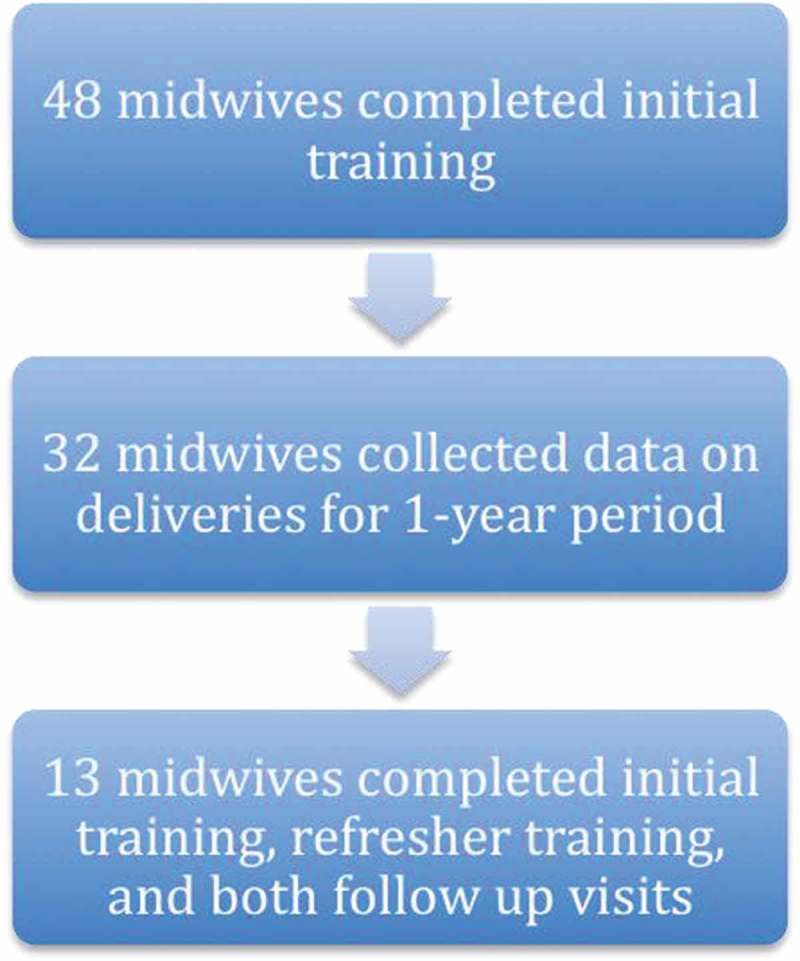
Number of midwives completing components of study.

A combined total of 48 students attended the initial trainings in the two sites. All trainees were women and all were Ghanaian Ministry of Health-certified midwives. Thirty-two of the midwives recorded data from deliveries during the subsequent year. Thirteen of the midwives completed all four of the following: initial training, refresher course at one year, OSCE at four months after initial training, and OSCE at four months after the refresher course (see Figures 2 and 3).

**Figure 4.**
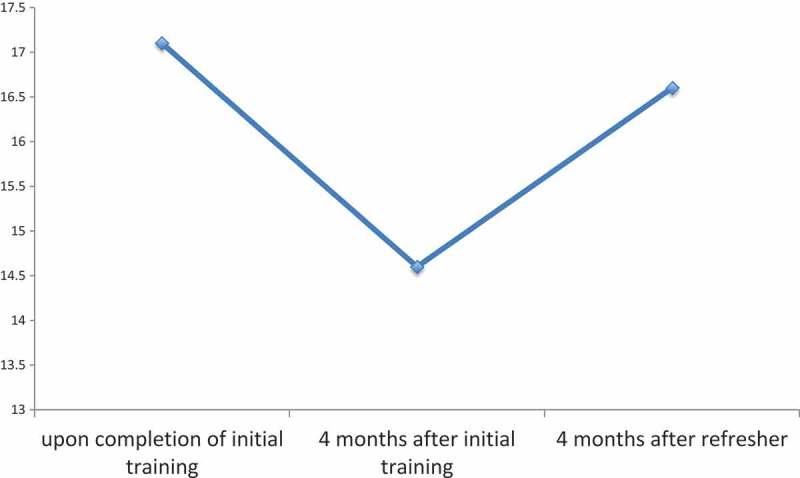
Midwives’ mean OSCE-B Score x/18.

**Figure 5.**
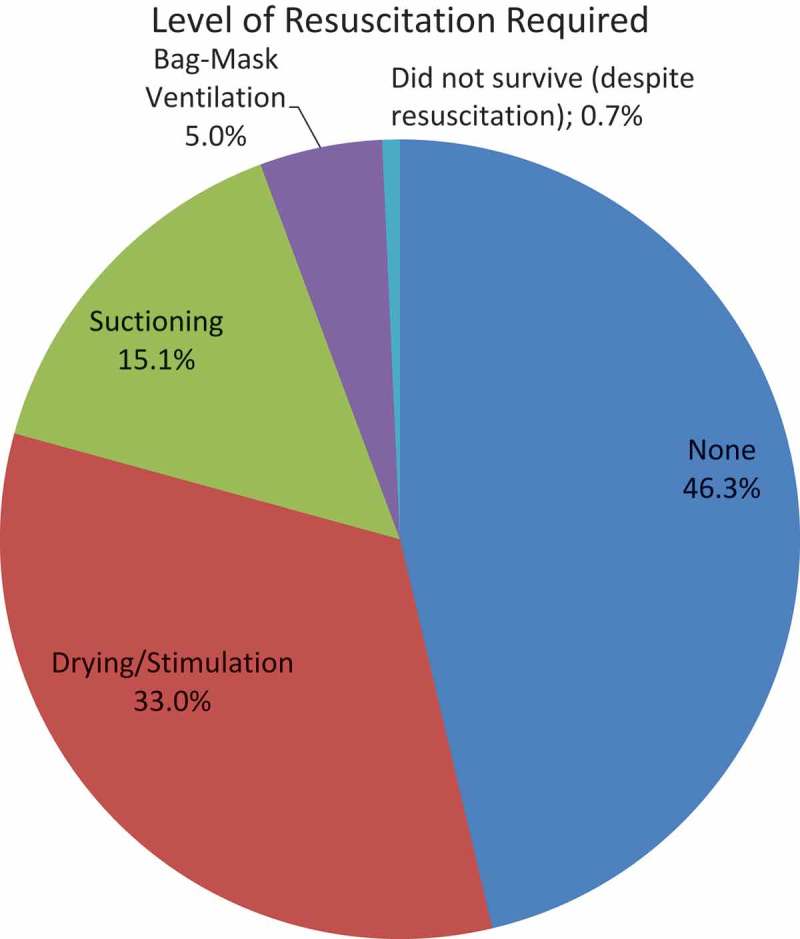
Level of resuscitation required.

Immediately following initial training, the mean OSCE score was 17.1/18 (94.9%). Four months later, the average score was 14.6/18 (81.2%, 95% CI for difference = −8.10% to −19.3%, p < 0.0001). Four months following the refresher course, the mean OSCE score was 16.6/18 (92.7%), an improvement of 11.5% from four months after the initial course (95% CI = 5.51% to 17.6%, p = 0.0013). There was no significant difference between the mean score after the initial training and the mean score four months after the refresher course (95% CI = −5.64% to 1.36%, p = 0.21) (see Figure 4). All 13 midwives passed the exam after the initial training, 11 of 13 passed four months later, and all 13 passed four months following the refresher.

The 32 midwives recorded information from 2,383 deliveries performed. 1,103 (46.3%) newborns were crying at birth before being dried. There were 786 (33.0%) who required only drying and/or stimulation; 359 (15.1%) required suctioning; 118 (5.0%) required bag-mask ventilation; 17 (0.71%) did not survive the first 24 hours of life (see Figure 5). In accordance with HBB teaching, we assume the 17 infants that did not survive were provided with all steps of resuscitation including bag-mask ventilation.

## Discussion

Simulation results achieved by the midwives trained by this HBB program suggest that training is associated with an improvement in students’ practical skills. Although the skills acquired through initial training diminish over time, our findings indicate the skills diminish less quickly when supplemented by a single two-day refresher course provided one year after the initial training. It is also likely that the midwives’ self-reflection after each delivery as a result of maintaining delivery logs also contributed to the delay in skills decay following the refresher when compared to the initial course, which has also been suggested by a study in Nepal [14].

The decline in skills we observed after the first four months is consistent with previous research [9,10]. The most effective method to refresh these skills, however, has yet to be determined. Several studies have documented either improvement on simulation exercise or in observed delivery room performance when students were provided with brief, frequent refresher training sessions, such as supervised daily practice [3], monthly 40 minute sessions [6], or visits to designated health centers every two weeks [4].

However, for an HBB student population of rural midwives staffing small clinics that cover a large geographical area, this type of ‘low intensity, high frequency’ training may be more expensive and less practical than more in-depth refresher courses administered less often. A large study in India demonstrated that a single course provided 6–8 months after initial training can also increase trainees’ simulation scores back to the high level initially attained [11], but did not evaluate retention past the immediate post-refresher period. To date, our results appear to be unique in demonstrating that performance following an HBB refresher course is more sustained than after the initial course alone. Additional research is needed to determine how long this improved performance will last, and exactly what frequency and intensity of training is most effective to maintain it. Ideally, consistent with the ‘training cascade’ suggested by the Helping Babies Survive initiative, foreign Master Trainers would transfer responsibility for refresher education to local Master Trainers, making the program more cost-effective and sustainable in the long term.

Regarding the impact of training on mortality in the first 24 hours of life, the result that 5.0% of the newborns in our study required bag-mask ventilation in order to survive is consistent with World Health Organization global estimates [2]. Since none of the midwives participating in this study had been trained in newborn resuscitation and did not own bag-mask ventilation devices before taking the Helping Babies Breathe course, it is reasonable to assume that the training prevented the death of at least some of the 118 newborns who were provided with BMV.

However, conclusively demonstrating that HBB training is associated with a decrease in mortality in a low-income, rural population is challenging. To date, the largest, most methodologically sound studies evaluating the HBB’s impact demonstrate a decrease in either newborn mortality rate (NMR) or fresh stillbirth rate (FSR) from before to after training mostly at large hospitals where data collection occurred before HBB was provided [11–15]. As discussed above, since the relative burden of newborn mortality is likely more significant in rural regions not regularly attended by physicians, birth attendants working in these areas are in greater need of resuscitation skills and therefore are a more ideal target population for HBB. This population, however, is the least likely to have accurate (if any) data recorded on deliveries before HBB training takes place. If it would delay education on interventions, such as bag-mask ventilation, that have clearly been proven effective in higher resource settings, prospectively collecting such data before providing this education may be unethical.

Therefore, although our study does not compare outcomes in a single, isolated population from before to after intervention, useful information can be inferred from comparisons to population estimates. The percentage of newborns in our study who did not survive the first 24 hour period was 0.71%. By comparison, a study in rural Ghana published in the *Bulletin of the World Health Organization* estimated that between 2007–2013, 1.7% of newborns died during the first 24 hours of life [16].

While conclusively determining the decrease in first 24 hour mortality due to HBB in this population may be impossible due to ethical concerns, our lack of location-specific data on newborn mortality from before training is nonetheless one of our study’s limitations. Another limitation is the loss to follow up of several midwives at various stages of the study. The fact that only 67% of trained midwives collected delivery data and only 27% completed all three practical evaluations for comparison may have introduced selection bias into our results.

A final limitation of this study is that the simulation examinations were administered and scored by four different individuals, without evaluating for inter-rater variation such as in a study from Sudan [10]. However, as the OSCE remains consistent across time and among all study subjects, and all evaluators received the same HBB Master Trainer course, it is not surprising that the Sudanese study found strong inter-rater agreement, and therefore this did not likely significantly alter our results.

Despite these limitations, our results suggest that providing an in-depth refresher course one year after HBB training is one effective method to solidify the skills learned by trainees, and when provided to the right population, these skills likely decrease neonatal deaths from birth asphyxia. Since evaluating outcomes among this population is challenging ethically and logistically, additional study is necessary to evaluate the reproducibility of these results. However, given the empowering nature of the education for midwives and the relatively low cost of the program [17], these results should complement existing findings demonstrating that when used appropriately, the Helping Babies Breathe program can be a beneficial and cost-effective intervention.

## Paper context

The Helping Babies Breathe program teaches basic newborn resuscitation skills to birth attendants in low-resource settings. This study found that when students were provided with an in-depth refresher course one year after the initial training, practical skills did not decay as quickly. Of 2,383 deliveries recorded, 5.0% newborns survived after receiving bag-mask ventilation. 0.71% did not survive 24 hours, compared with nationwide estimates of 1.7%, suggesting the program prevented deaths due to birth asphyxia.
